# Evaluation of the Proliferative Activity of Diffuse Large B-Cell Lymphoma (DLBCL) in Dogs with Respect to Patient Eligibility for Anthracycline-Based Chemotherapy

**DOI:** 10.3390/ani11041183

**Published:** 2021-04-20

**Authors:** Paweł Klimiuk, Wojciech Łopuszyński, Kamila Bulak, Adam Brzana

**Affiliations:** 1 Veterinary Diagnostic Laboratory VetDiagnostyka, 20-418 Lublin, Poland; 2 Sub-Department of Pathomorphology and Forensic Veterinary Medicine, Department and Clinic of Animal Internal Diseases, University of Life Sciences in Lublin, 20-601 Lublin, Poland; wojciech.lopuszynski@up.lublin.pl (W.Ł.); kamila.bulak@up.lublin.pl (K.B.); 3 Regional Veterinary Inspectorate in Opole, Regional Veterinary Laboratory, 45-836 Opole, Poland; a.brzana@wiw.opole.pl

**Keywords:** malignant lymphoma, dog, cell proliferation, chemotherapy, Ki67, topoisomerase IIα

## Abstract

**Simple Summary:**

Canine lymphomas usually have aggressive behavior, but respond well to chemotherapy. Despite proper diagnosis and numerous available therapeutic regimens, it is difficult to determine the prognosis and choose the optimal method of treatment in each individual patient. Diffuse large B-cell lymphoma is the most commonly diagnosed histological subtype of canine lymphoma and is treated with anthracyclines alone or in combination with other chemotherapeutics. A new diagnostic marker of prognostic and predictive value is topoisomerase IIα, which also constitutes a molecular target for anti-cancer drugs belonging to the group of topoisomerase IIα inhibitors including anthracyclines. Proliferative activity was estimated in samples of enlarged lymph nodes in dogs with diffuse large B-cell lymphoma based on mitotic count and immunohistochemical evaluation of topoisomerase IIα and Ki67 antigen expression with a view to qualifying patients for anthracycline-base chemotherapy. It has been shown that higher levels of topoisomerase IIα expression corresponded to a higher mitotic count but not to Ki67 index. These results indicate that an immunohistochemical evaluation of topoisomerase IIα expression can be used to develop a diagnostic-clinical protocol for the treatment of dogs with diffuse large B-cell lymphoma using anthracycline-based chemotherapy.

**Abstract:**

Different types of canine lymphoma respond differently to chemotherapy and have different prognoses. Diffuse large B-cell lymphoma (DLBCL) is the most common lymphoma in dogs. Topoisomerase II alpha (TOPIIα) protein has been shown to be a proliferation marker associated with prognostic significance. The aim of the study was to determine the relationship between TOPIIα expression, mitotic count (MC), and Ki67 antigen index in DLBCL in dogs, taking into account the applicability of these parameters to select the chemotherapy protocol with emphasis on the use of anthracycline drugs. Samples of formalin-fixed paraffin-embedded lymph nodes from 34 dogs with DLBCL were immunohistochemically labelled with anti-TOPIIα and Ki67. The number of positive cells and the intensity of the reaction were taken into account in order to assess TOPIIα expression. MC was estimated in the hematoxylin and eosin-stained slides in the area of 2.37 mm^2^. Positive association between TOPIIα and MC, but no association between TOPIIα and Ki67 was found. It can be concluded that the immunohistochemical determination of TOPIIα as a molecular target for drugs from the anthracycline group may be used in association with MC to establish a diagnostic-clinical protocol for selecting dogs with DLBCL for treatment with anthracycline drugs.

## 1. Introduction

Non-Hodgkin lymphomas are the most frequently diagnosed neoplasms of the hematopoietic system in dogs. They are ranked third amongst the most prevalent mammary and skin tumors [1,2,3]. The most commonly diagnosed histological subtype of canine lymphoma is diffuse large B-cell lymphoma (DLBCL) [4,5,6,7,8,9]. Most cases of canine lymphoma are diagnosed in clinical stage III, IV, or V, according to WHO criteria [10,11]. The basic diagnostic procedure in the case of suspected lymphoma is the cytological examination of the fine-needle aspirates from neoplastic lymph nodes. According to WHO guidelines, histopathological examination of a surgically removed lymph node supplemented with immunophenotyping and polymerase chain reaction (PCR) for antigen receptor rearrangement (PARR) is useful to definitively diagnose, establish prognosis, and in some cases guide treatment options [12,13,14,15]. The most common method for assessing the lymphoma phenotype is immunohistochemistry, while, in order to assess the malignancy and prognosis of the disease progression, cell proliferation indicators are taken into account, including the mitotic count (MC) and the assessment of the expression of the cell cycle dependent Ki67 protein [11,15,16,17]. Lymphomas are neoplasms with high chemosensitivity [18,19,20]. The chemotherapy of canine malignant lymphoma involves single or multi-drug protocols which allow for complete or partial remission and, in some cases, can extend a dog’s life up to 36 months [21,22,23,24,25]. At the same time, despite confirmed diagnosis and numerous available therapeutic regimens, it is difficult to determine the prognosis, choose the optimal method of treatment, and predict the response to treatment in each individual patient. Due to the fact that most cytostatic drugs affect actively dividing cells, the basic indicator of tumor chemosensitivity is the determination of the fraction of cells going through the cell cycle. Moreover, the information on proliferative activity allows prediction of the tumor’s aggressiveness and identification of cases with the prognoses of a quick relapse or the risk of metastasis. The side effects of chemotherapy drugs, such as nephrotoxicity, hepatotoxicity, and cardiotoxicity, as well as differentiated response to treatment, imply the need for a careful assessment of tumor cells’ susceptibility to treatment. Counting of cells demonstrating mitotic figures and the assessment of the expression of regulatory proteins, such as Ki67, is most commonly used to assess the proliferative activity of neoplasms [17,26]. A new proliferation marker of prognostic and predictive value is topoisomerase IIα (TOPIIα), which also constitutes a molecular target for some anti-cancer drugs belonging to the group of TOPIIα inhibitors. Topoisomerases are members of the family of enzymes involved in DNA metabolism, including transcription, recombination, replication, and chromosome segregation during cell division. TOPIIα is associated with the segregation of the newly replicated chromosome pairs, condensation and formation of the chromosome framework, and modification of the DNA superhelix [27,28,29,30]. TOPIIα expression is the highest in the G2/M phases of the cell cycle, and the lowest at the end of mitosis. The largest group of cytostatic drugs affecting this protein are anthracyclines, which, due to relatively good therapeutic effects, moderate adverse reactions, and low cost, are commonly used in veterinary oncology. The most commonly used drugs belonging to this group are doxorubicin and epirubicin [31,32,33,34,35]. These drugs create a cleavable complex consisting of the drug, TOPIIα, and DNA strand, blocking relegation and enzyme release, leaving the DNA with a permanent double strand break leading to cell apoptosis [30].

The aim of the study was to determine the relationship between TOPIIα expression and MC and Ki67 antigen index in DLBCL in dogs, taking into account the applicability of the determined parameters to establish the optimal chemotherapy protocol with emphasis on the use of anthracycline drugs.

## 2. Material and Methods

### 2.1. Material for the Study

The material for the study consisted of samples of enlarged popliteal lymph nodes surgically removed from 34 selected dogs of varied sex and age, with newly diagnosed, previously untreated lymphoma admitted to the clinics of Faculty of Veterinary Medicine in Lublin between January 2013 and December 2017. To be eligible for the study, dogs had to undergo a complete diagnostic evaluation, including history and physical examination, chest X-ray, and ultrasound of the abdominal cavity, as well as pathomorphological examination indicating the diagnosis of DLBCL. The surgical procedure with the use of propofol (Scanofol 10/mL, ScanVet, Gniezno, Poland) for anesthesia of dogs was performed according to the applicable rules, after obtaining the written consent of the owner.

### 2.2. Histology and Immunohistochemistry

Sections of lymph nodes were fixed in 10% buffered formalin with pH = 7.2 for 24 h and, after being dewaxed and rehydrated through a series of graded alcohol solutions in a tissue processor (Leica TP-20), were embedded in paraffin blocks. The 4 µm tissue sections were stained with hematoxylin and eosin (HE). Histologic classification followed the WHO criteria [6]. For immunohistochemical examination, the streptavidin–biotin–peroxidase complex technique was used (LSAB plus, horseradish peroxidase -HRP, K0690, Dako, Glostrup, Denmark). The lymphoma phenotype was determined using CD3 and CD79α antibodies. The cell proliferation activity was determined using Ki67 and TOPIIα antibodies. The primary antibodies used in the study are summarized in Table 1. 

Dewaxed and rehydrated sections were subjected to antigen retrieval. Target antigen retrieval for CD3 was performed by immersion in proteinase K for 10 min, whereas heat-induced epitope retrieval for CD79α, Ki67, and TOPIIα were performed by an automated pressure cooker in a citrate buffer of pH 6.0. The enzyme labelling the reaction site was horseradish peroxidase conjugated with streptavidin. Tetrahydrochloride-3,-3-diaminobenzidine (DAB) was used as a chromogen (SK-4100; Vector Laboratories, Peterborough, UK). The sections were counterstained with Mayer’s hematoxylin and covered in PERTEX (Histolab). For each assay, a double control system was used, i.e., method control and negative control. In the negative controls, the incubation with the primary antibody was replaced by incubation with mouse IgG serum under the same conditions of time and temperature. The positive control was the healthy unchanged tissue from canine tonsil. At each stage, two independent pathologists performed the assessment of the histopathological preparations and immunohistochemical reactions. A computer-assisted microscopic image analysis system was used to quantify the immunohistochemical parameters. The system included a light microscope (Nikon Eclipse E-600, Nikon Instruments, Tokyo, Japan) coupled with a digital camera (Nikon DS-Fi1, Nikon Instruments, Tokyo Japan) and a PC with image analysis software (NIS-Elements BR-2.20, Laboratory Imaging, Praha, Czech Republic). Lymphoma phenotype was determined by the percentage of expression of CD3 and CD79α molecules (membrane and cytoplasmic reaction) in neoplastic cells in 10 fields of view under 40x magnification (in the area of 2.37 mm^2^). B-cell lymphomas were diagnosed when more than 80% of cells expressed the CD79α antigen. T-cell lymphomas were excluded from the study. When evaluating the expression of the Ki67 antigen at 40x magnification, an index was calculated using the percentage of positive cells in 500 tumor cells. To assess TOPIIα expression, the scoring system proposed by Remmele et al. was used to evaluate the expression of estrogen receptors [36]. Based on the analysis of no less than 500 cells at 40x magnification, the number of stained cells was estimated and assigned a score, where 0 points corresponded to no positive cells, 1 point to 25% positive cells, 2 points to 26–50% positive cells, 3 points to 51–75% positive cells, and 4 points to over 75% positive; and the intensity of their staining, where 0 points—no reaction, 1 point—low staining intensity, 2 points—medium staining intensity, and 3 points—strong staining intensity. The final result was the product of points obtained from the assessment of both parameters, where 0 points meant no expression (-), 1 point meant very low expression (very low “+”), 2–4 points meant low expression (“++”), 5–7 meant medium expression (“+++”), and 8–12 (“++++”) meant high TOPIIα expression. MC was assessed in hematoxylin and eosin-stained sections by counting cells demonstrating mitotic figures in 10 fields of view at 40x magnification (in the area of 2.37 mm^2^).

### 2.3. Statistical Analysis

The obtained results were subjected to statistical analysis. The values of the measurable parameters were presented by means of the mean, median, lower and upper quartile, minimum and maximum values, and standard deviation; and for non-measurable ones, the number and percentage. The normality of the distribution of variables in the studied groups was checked using the Shapiro–Wilk normality test. The Student’s t-test was used to test the differences between the two groups, and in the case of failure to meet the conditions of its application, the Mann–Whitney test. Spearman’s rank correlation was used to assess the relationship between some of the variables. The level of significance was set at *p* < 0.05, indicating the existence of statistically significant differences or relationships. The database and statistical research were carried out on the basis of the Statistica 9.1 computer program (StatSoft, Cracow, Poland).

## 3. Results

Thirty-four dogs with DLBCL that met the admission criteria out of 67 dogs with diagnosed lymphoma were enrolled in the study. The dogs included in the study were 2.5 to 13 years old (median 6 years). The group consisted of 18 males (53%) and 16 females (47%). Mixed breed dogs: 10 (29.4%); German shepherd: 9 (26.5%); and Golden retriever: 4 (11.8%) were the most common breeds in this study. Stage III clinical progression was found in 20 dogs and stage IV in 14 dogs. Information regarding signalment, age, sex, breed, and stage of disease are presented in Table 2. 

**Figure 1:**
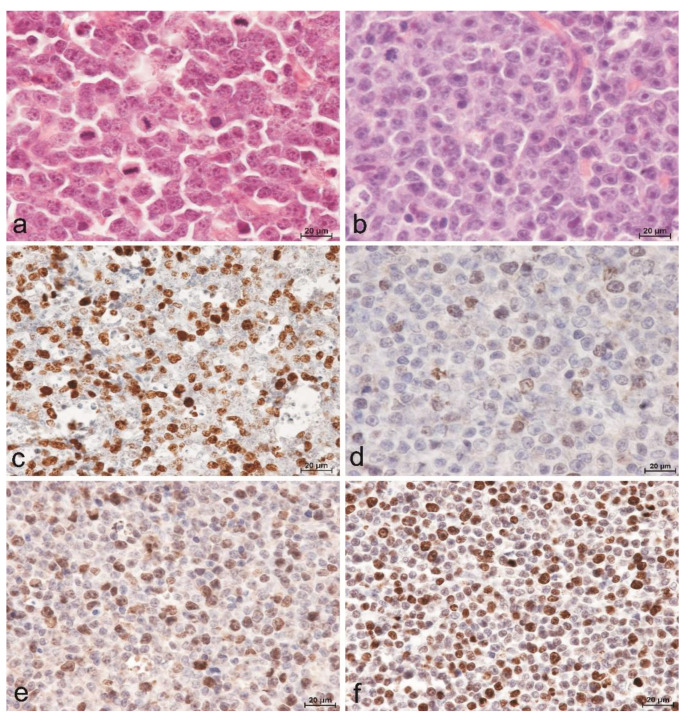
Lymphomas of diffuse architecture composed of large B cells. (**a**) Centroblastic subtype (DLBCL-CB) composed of uniform cells with round to oval vesicular nuclei with multiple small nucleoli and high mitotic rate. Hematoxylin and eosin staining. Bar = 20µm. (**b**) Immunoblastic subtype (DLBCL-IB) composed of large cells with round to oval nuclei with prominent single central nucleolus. Hematoxylin and eosin staining. Bar = 20µm. (**c**) DLBCL, centroblastic subtype with a high Ki67 index value. Indirect immunohistochemistry. Mayer’s hematoxylin counterstain. Bar = 20µm. (**d**) Low, (**e**) moderate, (**f**) high level of TOPIIα expression in DLBCL. Indirect immunohistochemistry. Mayer’s hematoxylin counterstain. Bar = 20 µm.

The diagnosis of DLBCL was made by evaluating cytological and histomorphological features in conjunction with immunohistochemical analysis to detect expression of CD79α and CD3 by the tumor cells. All dogs included in this study had an expression level of CD79α exceeding 80% of tumor cells, while the level of CD3 expression was less than 20% of tumor cells. The hallmark histomorphologic feature used to diagnose DLBCL was diffuse effacement of nodal architecture by sheets of neoplastic B-cells with scanty cytoplasm and predominantly large nuclei (≥2 red blood cells in diameter). The nuclei of these cells were round, cleaved, or indented. Twenty-eight (82.4%) dogs were further classified with centroblastic subtype (DLBCL-CB) based on the presence of multiple nucleoli (Figure 1a), while six (17.6%) dogs were classified with immunoblastic subtype (DLBCL-IB) based on dominance of a single central prominent nucleolus (Figure 1b). A positive reaction for the presence of Ki67 antigen (Figure 1c) and TOPIIα were evident in all cases with a diffuse granular nuclear pattern. Although in the case of TOPIIα, the variability in the reaction intensity and the number of positive cells were demonstrated (Figure 1d–f).

The value of the Ki67 antigen index ranged from 23.8% to 72.3%. The median value of Ki67 antigen index in the DLBCL-IB group was higher (41.8%) than in the group of DLBCL-CB (38.1%) with no statistical difference (Table 3). Higher expression of TOPIIα was observed at the periphery of the examined lymph nodes. The areas with the highest expression were selected for the assessment. Based on the established TOPIIα expression scoring system, the most numerous were cases with moderate TOPIIα expression (13 cases, 38.2%), followed by strong expression (9 cases, 26.5%). The total number of lymphomas with moderate to high expression of TOPIIα was 22, which accounted for 64.7% of cases in the study population. DLBCL with very low or low TOPIIα expression included 12 cases (35.3%) (Table 4). There was no statistically significant difference in TOPIIα expression between DLBCL-CB and DLBCL-IB lymphomas. In both groups, most cases were characterized with a moderate level of expression and accounted for 35.71% and 50%, respectively. The MC value ranged from 9 to 135 (median value = 24.5), with the value in DLBCL-CB group being significantly higher (median value = 29.5) compared to the DLBCL-IB group (median value = 15.0; U = 11.5; *p* < 0.001) (Table 3).

**Figure 2:**
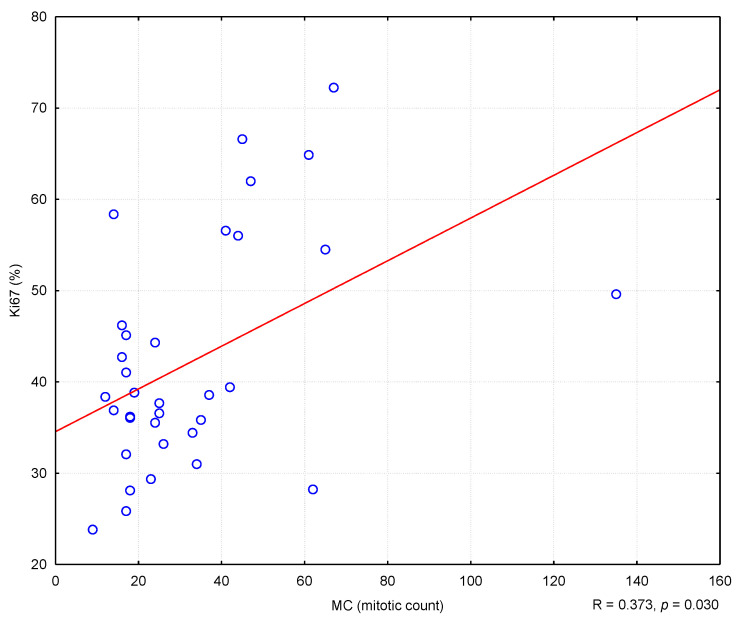
The correlation between Ki67 index and mitotic count (MC). R means Spearman’s rank correlation coefficient.

**Figure 3:**
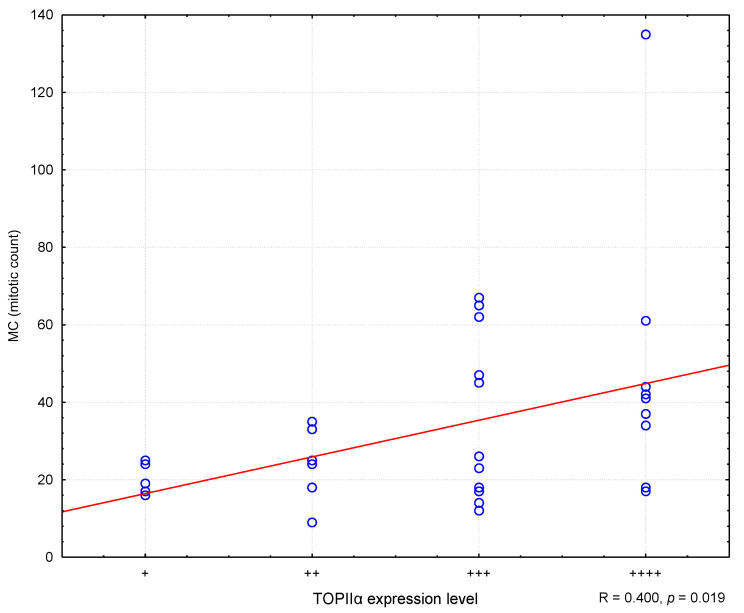
The correlation between TOPIIα expression and mitotic count (MC). R—Spearman’s rank correlation coefficient. “+” means very low expression, “++” means low expression, “+++” means medium expression, “++++” means high expression.

**Figure 4:**
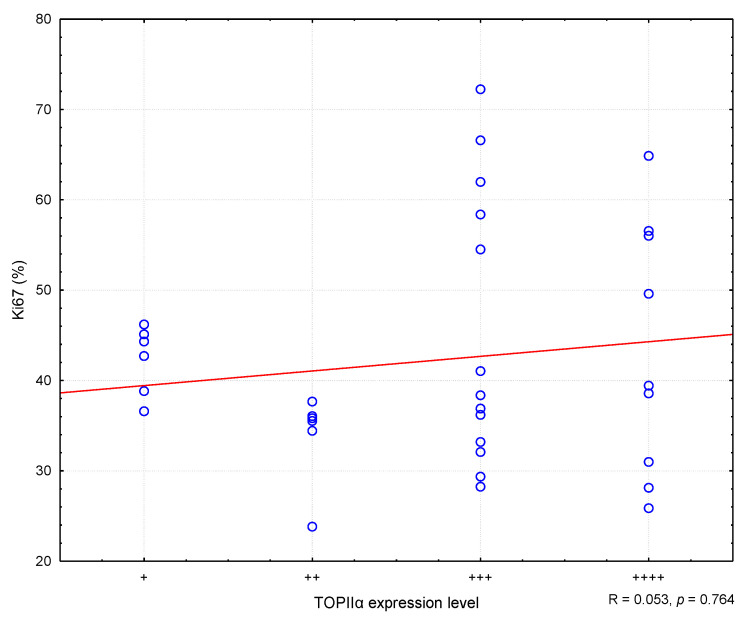
The correlation between TOPIIα expression and Ki67. R means Spearman’s rank correlation coefficient. “+” means very low expression, “++” means low expression, “+++” means medium expression, “++++” means high expression.

In the conducted study, it was found that higher MC values corresponded to higher Ki67 antigen index values, and the correlation was statistically significant (R = 0.373; *p* = 0.030) (Figure 2). Similarly, higher levels of TOPIIα expression corresponded to higher MC values (R = 0.400; *p* = 0.019) (Figure 3). However, no statistically significant correlation was found between the level of TOPIIα expression and the Ki67 values (R = 0.053; *p* = 0.764) (Figure 4). 

For the purposes of statistical analysis, lymphomas with very low and low TOPIIα expression (12 cases) were grouped, as well as lymphomas with moderate and high TOPIIα expression (22 cases). There were no statistically significant differences in the Ki67 and MC values between the group of lymphomas with very low and low TOPIIα expression and the group with moderate and high TOPIIα expression (Table 3).

## 4. Discussion

The aim of the study was to assess the cellular proliferation in DLBCL in dogs based on the expression of TOPIIα and Ki-67 antigen, and to determine the MC value in terms of patient qualification for chemotherapy with anthracyclines. DLBCL is the most commonly diagnosed lymphoma in dogs, accounting for approximately 30% of all lymphomas found in this species, most of which are the centroblastic subtype (DLBCL-CB), while the immunoblastic subtype (DLBCL-IB) is less prevalent [4,11,37]. This correlation was confirmed in the conducted study. In the vast majority of cases, the disease occurred in middle-aged (6 years old) large breed dogs (88%), most of which were mixed breed (29.4%), German Shepherd (26.5%), or Golden Retriever (11.8%). Most of the studies did not show a significant influence of breed, age, and gender on the risk of lymphoma development or on the course of the disease and prognosis regarding survival time and response to treatment [33,38]. Although one of the studies demonstrated increased susceptibility to the occurrence of lymphoma in unsterilized females and males, it has not been unequivocally confirmed in other studies [33]. In European countries, the breeds predisposed to lymphoma include Doberman, Rottweiler, Boxer, and Bernese mountain dogs, where boxers tended to develop T-cell lymphomas while Rottweilers had a high prevalence of B-cell lymphomas [39]. In our study, mixed-breed and German Shepherd dogs prevailed, which is undoubtedly related to the composition of the dog population in the region the animals came from and reflects the differences in the results of epidemiological studies [37,39]. Apart from the histological type and immunophenotype, the parameters determining the proliferative activity of cells, i.e., MC and Ki67 antigen index are the most frequently mentioned among the important prognostic factors in lymphomas in dogs. Despite the limitation of the study to only one type of lymphoma, the obtained results show considerable variation in the MC value and Ki67 antigen index in individual animals. The median MC value was 24.5 (9–135); whereby, in individuals with the centroblastic subtype, it was significantly higher (median = 29.5) than in those with the immunoblastic subtype (median = 15.0). The obtained mean values of MC are higher than those presented in the studies conducted by Valli et al. (DLBCL-CB 15.4; DLBCL-IB 13.8) [6]. The median Ki67 antigen index value was 38.5%. There was no statistically significant difference in either the median values of the Ki67 index, nor the level of TOPIIα expression between the respective subtypes of lymphoma (Table 3). The conducted study demonstrated a correlation between MC and Ki67 (*p* = 0.030) in the DLBCL group. Individuals with a higher MC value had a higher Ki67 antigen index. This correlation is due to the fact that the expression of the Ki67 antigen was found in all cells undergoing the cell cycle, so with the increase in the number of cells in the mitotic phase, the increase in the Ki67 value seems justified.

Another parameter that could potentially be used to assess the proliferative activity is the level of TOPIIα expression, which is also a molecular target for cytostatic drugs from the anthracycline group (mainly doxorubicin). Medical studies on the predictive and prognostic role of TOPIIα expression in humans have been conducted for many years [40,41,42,43,44,45]. Researchers are especially interested in the expression of this protein in breast cancer in women. [27,28,29,42]. The reason for this interest is the fact that doxorubicin is one of the main drugs used in the treatment of breast cancer in women [28]. It is surprising, however, that despite the fairly widespread use of doxorubicin in the treatment of various types of tumors in animals, including lymphomas, no studies have been carried out to date to determine the level of expression of this protein in individual tumors. In the conducted studies, the TOPIIα expression was assessed using a proprietary scheme, taking into account both the number of positive cells and the intensity of their staining. This scheme was used due to doubts raised by many authors regarding the lack of a uniform scheme allowing objective assessment and interpretation of the level of TOPIIα expression. The most commonly expressed problem was in regard to the need to establish the so-called cut-off point, i.e., determining what level of TOPIIα expression was considered as low, medium, or high. Most authors have only described the observed correlations between the TOPIIα expression and response to treatment, without quantifying the level of the expression. Hajduk et al., examining the level of TOPIIα expression in breast cancer cells, assumed that the lack of expression can be confirmed when the number of “positive” cells did not exceed 5%, low expression with 6–30%, moderate expression with 31–60%, and high expression with more than 60% of stained cells [27]. Pentheroudakis et al., examining the level of TOPIIα expression in DLBCL in humans, estimated that lymphomas with high TOPIIα expression are those in which positive cells constitute more than 80% of all cells. The remaining ones are lymphomas with low (less than 80%) expression of the protein [40]. In our study, positive expression of TOPIIα was observed in all the lymph node samples, but with varying intensity. Nevertheless, the vast majority of cases were characterized with high and medium expression (22 dogs: 64.7%), and cases with low and very weak expression constituted a minority (12 dogs: 35.3%). In similar studies carried out in people with DLBCL, high expression of TOPIIα was noted in 91% of patients [40]. The studies demonstrated that the level of TOPIIα expression positively correlated with the mitotic index (*p* = 0.019), but no significant correlation was found between the Ki67 antigen and TOPIIα values. 

All the parameters assessed by the authors allow the estimation of the proliferative activity of neoplastic cells, but they have a variable prognostic value regarding the determination of the patient survival time and predictive value in the context of choosing an appropriate therapy regimen. The most frequently used protocol in the treatment of canine lymphoma is CHOP (Cyclophosphamide, Hydroxydaunorubicin, Oncovin, and Prednisolone) [7,20,21,22]. The use of multi-drug protocols is important and justified, as each drug has an effect on cells at a different stage of the cell cycle. This allows the achievement of the best results in the form of complete or partial remission and longer overall survival. This protocol has been found to be most effective in patients with high-grade lymphomas, i.e., those with high MC. It was demonstrated that the higher the MC value, the shorter the survival time of dogs with DLBCL. At the same time, a higher MC value resulted in better treatment response, including the achievement of complete remission. Therefore, some authors considered MC to be the main prognostic parameter in canine lymphomas [16]. The role of Ki67 antigen expression as a prognostic factor in canine lymphomas is ambiguous. Kiupel et al. argued that Ki67 assessment has no statistically significant prognostic value in lymphomas in dogs [16]. According to the above-mentioned authors, the Ki67 antigen index allows the determination of the percentage of cells in the cell cycle, but it does not make it possible to determine the length of this cycle, i.e., it does not allow the assessment of the dynamics of the neoplastic process. Positive expression is observed in both cells with a cell cycle duration of 2 days and cells with a cell cycle duration of 30 days. On the other hand, a study by Sierra Matiz et al. demonstrated that in the group of dogs with DLBCL characterized with low Ki67 antigen expression, the survival times were significantly longer than in animals with high expression of this protein. [17]. According to the same authors, dogs with high Ki67 index demonstrated a much better response to treatment than those with low Ki67 antigen activity. The studies showed no significant differences in survival times and response to treatment in dogs with low and high MC values. The assessment of the prognostic value of TOPIIα expression in animals is the most challenging. The reason is the lack of any studies describing the behavior of this protein in animal tumors. Therefore, the results obtained in studies conducted in humans should be considered as a point of reference [40,41,42]. TOPIIα belongs to enzymes involved in the processes related to cell division and DNA metabolism [27,28,29,30]. At the same time, TOPIIα is an enzyme that served as a molecular target for cytostatic drugs from the anthracycline group (the most frequently used drug in this group is doxorubicin, which constitutes a part of the multi-drug CHOP regimen used in the treatment of lymphoma) [21,22,23,24]. Its expression reaches its peak in the G2/M phase, i.e., just before cell division. This explains the statistically significant correlation observed between the level of TOPIIα expression and the MC value in our study, and at the same time indicates that these two parameters may serve as significant prognostic indicators in selecting patients for anthracycline therapy. However, the authors of the article did not find any statistically significant relationships between the expression of TOPIIα and Ki67, which, combined with the observations of Kiupel et al., indicates the existence of differences in the expression of both proteins in the cell cycle [16]. On the other hand, in studies conducted by Pentheroudakis et al. and Korkolopoulou et al. on lymphomas in humans, a positive correlation was found between the expression of TOPIIα and Ki67 [40,41]. However, in the cited studies, only the percentage of cells showing a positive reaction was considered, without grading its intensity. Moreover, when comparing the same lymph node samples, it was noticed that all TOPIIα expressing cells also expressed Ki67, but not all Ki67 expressing cells expressed TOPIIα. Thus, it can be assumed that a high level of the Ki67 protein does not always indicate high TOPIIα expression. Considering that TOPIIα is a molecular target for drugs from the anthracycline group, it can be concluded that the differences in protein expression observed in our study in different individuals with DLBCL causes different responses to treatment with TOPIIα inhibitors. The obtained results indicate potentially greater chemosensitivity of lymphomas with high TOPIIα activity to the action of chemotherapeutic agents being the enzyme inhibitors. The fact that the conducted studies showed a relatively low degree of correlation between MC and Ki67 antigen index and between MC and TOPIIα expression, with no correlation between Ki67 antigen index and TOPIIα expression, indicates that the assessment of TOPIIα expression should be used as a key potential predictive indicator for anthracycline-base therapy. This is reflected in clinical observations in humans. According to Hajduk et al., in patients with high TOPIIα expression, the response to treatment with doxorubicin was better than in patients with low expression of this protein. Additionally, it was found that the overall survival of patients with low TOPIIα expression treated with doxorubicin was significantly lower than in the case of patients with high TOPIIα expression [27]. In human DLBCL patients with high TOPIIα expression, a much better response to treatment using the CHOP protocol was observed, however, it did not improve the patients’ overall survival [40]. The preliminary results of the authors’ clinical observations indicate that dogs with DLBCL and with high TOPIIα expression respond better to anthracycline-based therapy, which is manifested by a higher percentage of complete remission and a longer remission compared to dogs with DLBCL and low TOPIIα expression. However, before the studies are completed, it would be unjustified to draw any conclusions regarding the correlation between TOPIIα expression and the clinical effects of anthracycline-based therapy in dogs with DLBCL, despite the initial promising results.

## 5. Conclusions

The obtained results indicate that the immunohistochemical assessment of the TOPIIα expression may be used to develop a diagnostic clinical protocol for the treatment of dogs with diffuse large B-cell lymphoma using anthracycline-based chemotherapy. The limitations resulting from the small number of patients in our study influences statistical analysis, and the lack of standardized uniform schemes for assessing the expression of individual cell-cycle proteins imply the need for further studies in this area, in particular, clinical studies on a large population of animals, aimed at confirmation of correlation between the level of TOPIIα expression and the other parameters of proliferative activity, as well as the therapeutic effects of treating patients with TOPIIα inhibitors. 

## Figures and Tables

**Table 1:** Primary antibodies, resources, and dilution used in immunohistochemistry.

Antibody (Anti-)	Type and Clone	Manufacturer	Dilution
CD3	polyclonal, A0452	Dako, Glostrup, Denmark	1:300
CD79αc	monoclonal, HM57	Dako, Glostrup, Denmark	1:100
Ki67	monoclonal, MIB-1	Dako, Glostrup, Denmark	1:100
TOPIIα	monoclonal, Ki-S1	Dako, Glostrup, Denmark	1:200

CD3; T—lymphocyte marker, CD79α; B—lymphocyte marker, Ki67—cell proliferation marker, TOPIIα—topoisomerase IIα.

**Table 2:** Baseline characteristics of patient population.

Parameter		*n* = 34
Age	Average: 6.6 (median = 6.0, range: 2.5–13) SD ± 2.9	
Sex	Male (neutered male)	18 (3)
	Female (spayed female)	16 (9)
Breed	Mixed-breed	10
	German shepherd	9
	Golden retriever	4
	Boxer	2
	Dog de Bordeaux	2
	French bulldog	2
	Miniature schnauzer	2
	Rottweiler	2
	Bernese mountain dog	1
Clinical stage	III	20
	IV	14

**Table 3:** Comparison of the mitotic count (MC) and the Ki67 index values depending on the type of lymphoma and TOPIIα expression.

Parameter	Group	*n*	M	Me	Min	Max	Q1	Q3	SD	U	*p*
MC	DLBCL	34	32.85	24.5	9	135	17	42	24.3		
	CB	28	36.82	29.5	14	135	18.5	44.5	25.04	11.5	<0.001
	IB	6	14.33	15	9	18	12	17	3.39		
Ki67	DLBCL	34	42.24	38.47	23.83	72.23	34.43	49.61	12.44		
	CB	28	42.43	38.12	25.87	72.23	33.82	52.06	12.82	79	0.843
	IB	6	41.35	41.75	23.83	58.37	36.2	46.21	11.57		
Ki67	TOPIIα									–1.459 *	0.154
	expression +/++	12	38.1	37.13	23.83	46.21	35.68	43.52	6.11		
	expression +++/++++	22	44.5	39	25.87	72.23	32.09	56.56	14.45		
MC	TOPIIα									82.5	0.074
	expression +/++	12	21.75	21.5	9	35	16.5	25	7.42		
	expression +++/++++	22	38.91	35.5	12	135	17	47	28.11		

DLBCL—diffuse large B-cell lymphoma, CB—centroblastic subtype, IB—immunoblastic subtype, *n*; number of cases, M—mean, Me—median, Min—minimum value, Max—maximum value, Q1—lower quartile, Q3—upper quartile, SD—standard deviation, U—U Mann–Whitney test result, *p*—statistical significance, and * t-Student test analysis.

**Table 4:** Comparison of TOPIIα expression in DLBCL-CB and DLBCL-IB lymphomas.

TOP II Expression Level	DLBCL	
	CB	IB
+	4	2
	14.29%	33.33%
++	5	1
	17.86%	16.67%
+++	10	3
	35.71%	50.00%
++++	9	0
	32.14%	0.00%
Total	28	6
Mean rank	18.63	12.25
Statistical analysis	U = 52.5 *p* = 0.159	

DLBCL—diffuse large B-cell lymphoma, CB—centroblastic subtype, IB—immunoblastic subtype.

## Data Availability

The datasets used and analyzed in the current study are available from the corresponding authors on reasonable request.
